# Ultrasound localization microscopy of renal tumor xenografts in chicken embryo is correlated to hypoxia

**DOI:** 10.1038/s41598-020-59338-z

**Published:** 2020-02-12

**Authors:** Matthew R. Lowerison, Chengwu Huang, Fabrice Lucien, Shigao Chen, Pengfei Song

**Affiliations:** 1 Beckman Institute for Advanced Science and Technology, University of Illinois at Urbana-Champaign, Urbana, IL USA; 2 Department of Electrical and Computer Engineering, University of Illinois at Urbana-Champaign, Urbana, IL USA; 3 Department of Radiology, Mayo Clinic College of Medicine and Science, Mayo Clinic, Rochester, MN USA; 4 Department of Urology, Mayo Clinic College of Medicine and Science, Mayo Clinic, Rochester, MN USA

**Keywords:** Cancer imaging, Tumour angiogenesis, Renal cancer

## Abstract

Ultrasound localization microscopy (ULM) permits the reconstruction of super-resolved microvascular images at clinically relevant penetration depths, which can be potentially leveraged to provide non-invasive quantitative measures of tissue hemodynamics and hypoxic status. We demonstrate that ULM microbubble data processing methods, applied to images acquired with a Verasonics Vantage 256 system, can provide a non-invasive imaging surrogate biomarker of tissue oxygenation status. This technique was applied to evaluate the microvascular structure, vascular perfusion, and hypoxia of a renal cell carcinoma xenograft model grown in the chorioallantoic membrane of chicken embryos. Histological microvascular density was significantly correlated to ULM measures of intervessel distance (R = −0.92, CI_95_ = [−0.99,−0.42], p = 0.01). The Distance Metric, a measure of vascular tortuosity, was found to be significantly correlated to hypoxyprobe quantifications (R = 0.86, CI_95_ = [0.17, 0.99], p = 0.03). ULM, by providing non-invasive *in vivo* microvascular structural information, has the potential to be a crucial clinical imaging modality for the diagnosis and therapy monitoring of solid tumors.

## Introduction

The vascular supply within tissue is a critical micro-environmental factor that informs tissue oxygenation, development, nutrient delivery, and metabolic demand. Quantified metrics of vascular supply have the potential to inform a broad spectrum of pathological and mal-adaptive tissue states. For example, regionalized hypoxia has been linked to impaired tissue health and poor patient outcomes across multiple disease sites including cancer^1^, stroke^2,3^, and heart disease^4^. Pathological angiogenesis, which arises from unbalanced pro-angiogenic and anti-angiogenic cytokine signaling^5^, is a hallmark of cancer^6^ which precludes the development of fully functional vessel networks within solid tumors. The vascular supply of cancerous tumors is characteristically chaotic, with a topographically disordered and inefficient capillary structure yielding disorganized and intermittent blood flow^7^. A consequence of this poor and aberrant vascular perfusion is persistent and regionalized intratumoral hypoxia, a critical tumor micro-environmental feature that has been correlated with clinical resistance to conventional cytotoxic therapies such as chemotherapy^8^, radiotherapy^9^, and immunotherapy^10^. Tumor hypoxia has also been shown to select for resistance to apoptosis^11^, drive genetic instability^12,13^, and increase the risk of both local invasion and metastatic spread^14^. Despite hypoxia exhibiting a profound impact on patient outcome, it remains a seldom explored clinical biomarker due to the highly invasive techniques that are currently used to quantify tissue oxygenation status. There is a need for non-invasive clinical imaging technologies that can inform the vascular supply status of deep tissues with the intent of identifying intratumoral hypoxia.

Optical imaging modalities, such optical coherence tomography (OCT) and laser Doppler, offer excellent imaging resolution that can provide capillary-level microvascular detail, but these techniques are limited to shallow imaging depths which impede clinical utility^15,16^. Dynamic contrast-enhanced magnetic resonance imaging (DCE-MRI) has demonstrated considerable clinical use for tumor perfusion imaging^17^, however, these perfusion measurements suffer from low temporal resolution, and will vary depending on the specific imaging sequences and pharmacokinetic models used for evaluation^18^. Likewise, perfusion computed tomography (CT) has had substantial clinical use in tumor vasculature characterization^17^, but is inevitably burdened by patient exposure to ionizing radiation and contrast agent nephrotoxicity^19^.

Contrast-enhanced ultrasound (CEUS) is a widely available and cost-effective clinical imaging modality that offers vascular perfusion imaging with clinically relevant penetration depths^20^, while maintaining ultrasound’s safety and lack of ionizing radiation. Conventionally the technique cannot resolve features that are below the diffraction limit of ultrasound and quantification metrics of tissue vascularity suffer from multiple sources of variability^21^. Recent developments in imaging technologies and techniques have allowed for the localization and tracking of individual contrast microbubbles *in vivo*, such as ultrasound localization microscopy or ULM^22–25^, permitting the reconstruction of higher resolution images to reveal tissue microvascular structure. This processing technique retains the imaging penetration, non-invasiveness, and safety profile of clinically used contrast-enhanced ultrasound, achieving a substantial improvement in spatial resolution in exchange for increased imaging acquisition times. Resolving microvascular features is of paramount importance for informing tissue vascular supply and oxygenation status, as the main site of gas- and nutrient-exchange occurs at the capillary bed. This revolutionary technological advance can be potentially leveraged to provide noninvasive quantitative measures of tissue hemodynamics and oxygenation within clinically-relevant decision times, thereby permitting tailored therapy selection based on tumor hypoxia status.

The application of ULM imaging for the characterization of intratumoral vasculature has been previously investigated by a number of research groups. Lin *et al*.^26^ used ULM to image tumor angiogenesis in a rat model of fibrosarcoma. They found a significant increase in the Distance Metric (DM), an established quantified metric of vascular tortuosity^27^, in tumor bearing rats in comparison to control. Opacic *et al*.^28^ performed an extensive analysis on tumor characterization with ULM imaging on mouse tumor xenografts, and validated their findings with gold-standard contrast-enhanced *ex vivo* microCT and CD31 immunohistochemistry. They produced a tumor classifier on the basis of ULM morphological and physiological parameters to discriminate intratumoral vascular phenotypes. They then applied their motion-model ULM technique to a clinical proof of concept study on patients with breast cancer, and then later extended this clinical pilot study in a report by Dencks *et al*.^29^. Although ULM has shown great promise in characterizing and classify tumor microvasculature, the application of ULM for measurement tumor hypoxia is relatively unexplored, which is surprising given that conventional CEUS has been shown to provide surrogate biomarkers for intratumoral oxygenation^30^.

In this paper we explore the utility of ULM imaging for providing a surrogate imaging biomarker for intratumoral hypoxia in a renal cell carcinoma xenograft model (ATCC CRL-2947, *Mus musculus* renal adenocarcinoma) grown in the chorioallantoic membrane (CAM) of chicken embryos^31^. The chicken embryo tumor xenograft model provides an ideal experimental model for ULM imaging given that there is minimal tissue motion, long microbubble recirculation times, and the tumor environment can be controlled. Ultrasound images were acquired from six contrast microbubble injected CAMs using a Verasonics Vantage 256 system and L35-16vX transducer. Super resolution microbubble localization and velocity maps were reconstructed at a 5 µm axial/lateral resolution. ULM analysis was performed on this dataset, yielding morphometric and perfusion-based quantifications of tumor vasculature. These results were compared with gold-standard fluorescent histology quantifications of microvascular density and hypoxia. Histological microvascular density, as measured by the mean fluorescent intensity of intravascular rhodamine lectin, was significantly correlated to ULM measures of intervessel distance. We also found that vascular tortuosity, as measured by DM on ULM images, was significantly correlated to hypoxyprobe quantifications. ULM has the potential to be a crucial clinical imaging modality for the diagnosis and therapy monitoring of solid tumors by providing non-invasive *in vivo* microvascular structural information. This modality is inexpensive and does not use ionizing radiation, warranting frequent clinical follow-up exams.

## Results

### The ex ovo CAM tumor model is highly accessible for ULM acquisitions

**Figure 1:**
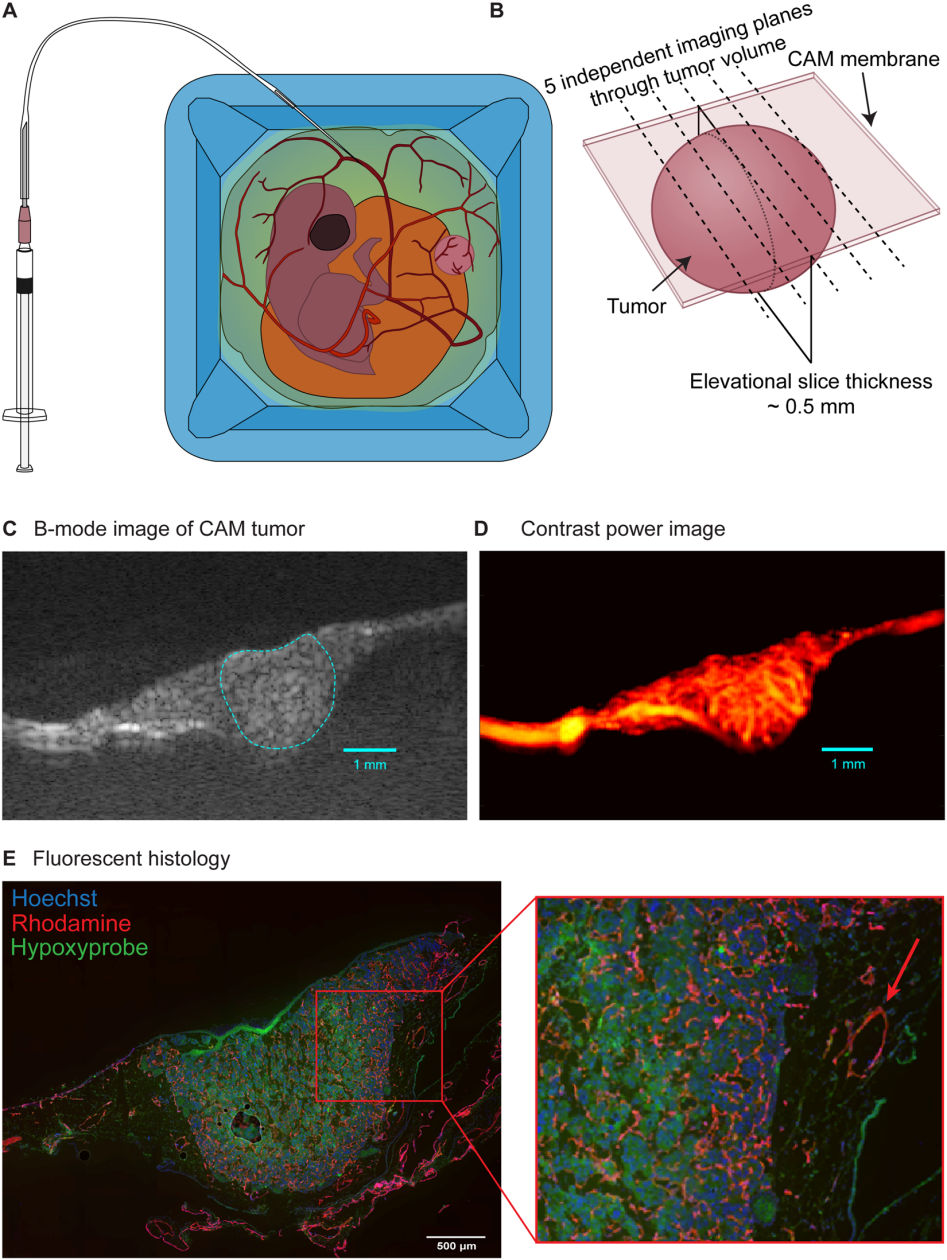
Vascular imaging of CAM-engrafted Renca tumors. (**A**) The *ex ovo* chicken embryo model has readily accessible surface vasculature, facilitating the injection of precise volumes of microbubble contrast agent. Renca cells engrafted into the CAM surface result in spheroidal tumor masses that are consistently hyper-vascularized. (**B**) The spheroidal Renca tumors were imaged with ultrasound at five distinct imaging planes to volumetrically sample these renal tumor xenografts. Each imaging plane was imaged using 15 angle plane-wave compounding with a Vantage 256 system (L35-16vX transducer), for a total acquisition length of 3600 frames (7.2 s). (**C**) B-mode image of example tumor, generated by compounding plane-wave acquisitions, demonstrates a high SNR and well characterized tumor margin. (**D**) Conventional contrast-enhanced ultrasound image of example tumor confirms the high degree of vascularization typical of CAM-engrafted Renca tumors. Nominal imaging resolution was 50 µm axial and 100 µm lateral. (**E**) Fluorescent histology section, taken from the example tumor in (**D**), demonstrates both a high peripheral tumor vascularization (red signal) as well as intratumoral hypoxia (green signal).

As previously reported^31^, the Renca cell line consistently produces spheroid tumors that are highly vascularized when engrafted into the CAM of chicken embryos. All seven embryos inoculated with Renca cells produced tumors at endpoint; however, one embryo died after imaging. The exposed chorioallantoic membrane provides ample candidate vessels for the intravascular injection of precise volumes of microbubble contrast agents (Fig. 1A). The ease of locating tumor masses permitted a systematic sampling of the tumor volume. We performed ULM imaging on five evenly spaced tumor cross-sections for every Renca tumor (Fig. 1B). Compounded plane-wave imaging of these tumors resulted in B-mode images with a high SNR that demonstrated well-characterized tumor margins, permitting manual delineation of the tumor boundary (Fig. 1C). Contrast-enhanced power processing confirmed that these tumors were highly vascularized in the tumor center, tumor periphery, and in peritumoral tissues, as demonstrated in Fig. 1D. These findings were compared with fluorescent histology (Fig. 1E) which revealed extensive tumor microvasculature (red signal), particularly in the tumor periphery. The red arrow in Fig. 1E highlights the vessel lumen of a feeding arteriole. We found that the tumor center generally had less microvascular signal than the tumor periphery and demonstrated a corresponding high degree of hypoxyprobe expression (green signal). This suggests that the Renca tumors in this study were hypoxic and therefore exhibiting reduced intratumoral vascular supply, a feature that was not evident on conventional CEUS processing.

### Super-resolution ULM detects capillary blood flow in CAM tumors

**Figure 2:**
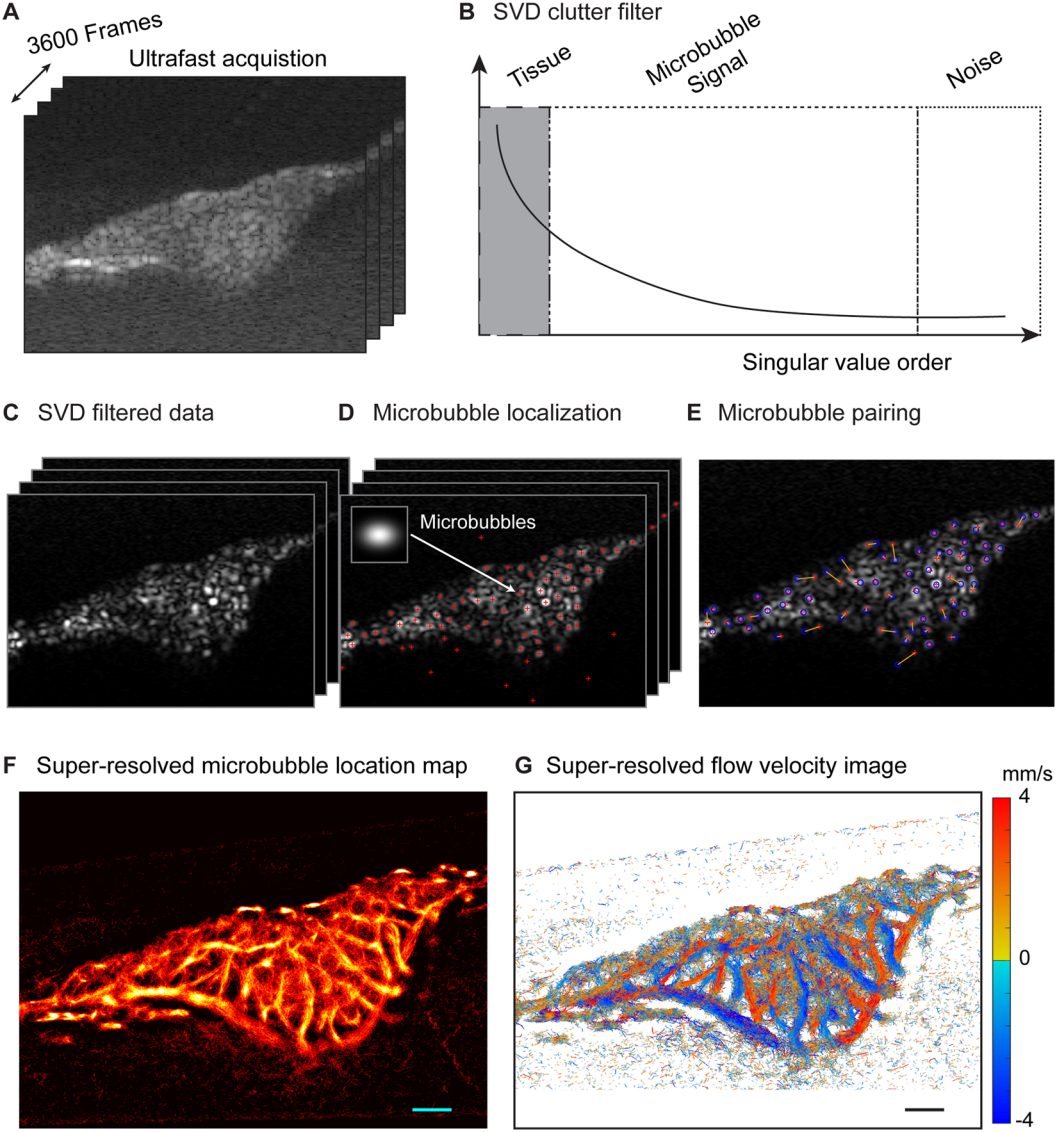
Microbubble localization and tracking. (**A**) IQ data (3600 frames) was represented as a three-dimensional matrix by stacking each B-mode image frame together along a ‘slow-time’ dimension. (**B**) The resulting imaging volume was reshaped into a 2D Casorati matrix in a column-wise manner, and a singular value decomposition (SVD) was used to remove the highly spatiotemporally coherent tissue background (low singular value orders). (**C**) An inverse SVD was performed to recover a three-dimensional imaging volume that contained predominantly microbubble signals. A microbubble PSF was convolved with each frame of the dataset shown in (**C**) to identify the positions of potential microbubble locations. (**D**) Representative image stack of localized microbubble centroids, after a local maximum search. There are some false positive microbubbles identified in this first pass of localization processing. (**E**) Bipartite graph-based filtering was used to pair microbubbles along several frames of data. This process reduces the number of false positive microbubble detections by eliminating low-confidence events. (**F**) Representative microvascular structure map from an example tumor. (**G**) Blood-flow velocity map for the same representative tumor.

Consistent with the findings of other investigators^22,32^ we were able to reconstruct ULM images at a < 10 µm pixel size, which may permit the localization of microvascular scale features. Most of the tissue background present in the B-mode cineloops (Fig. 2A – generated by compounding plane-wave acquisitions, see **Methods**) was eliminated through spatiotemporal filtering via singular value decomposition (SVD) processing^31,33^ (Fig. 2B), resulting in imaging stacks of predominantly microbubble signal (Fig. 2C). The locations of all the microbubbles in these filtered imaging datasets could be identified by cross-correlating the image data with a simulated point-spread-function of an individual microbubble that was based on previous experimental data (Fig. 2D, inset). The centroids of each microbubble could then be localized to a sub-pixel accuracy via a local maxima search (Fig. 2D). We detected an average of 57 microbubbles per frame, or 28,500 microbubbles per second. Microbubble tracks were then reconstructed via a bipartite graph-based pairing algorithm (Fig. 2E)^34^ and Kalman filtering^35^. The resulting microbubble tracks could then be accumulated to produce microvascular structure maps (Fig. 2F), and the frame-to-frame pairing information could be used to generate blood-flow velocity maps (Fig. 2G).

### Super-resolution quantifications are less variable than diffraction limited CEUS

**Figure 3:**
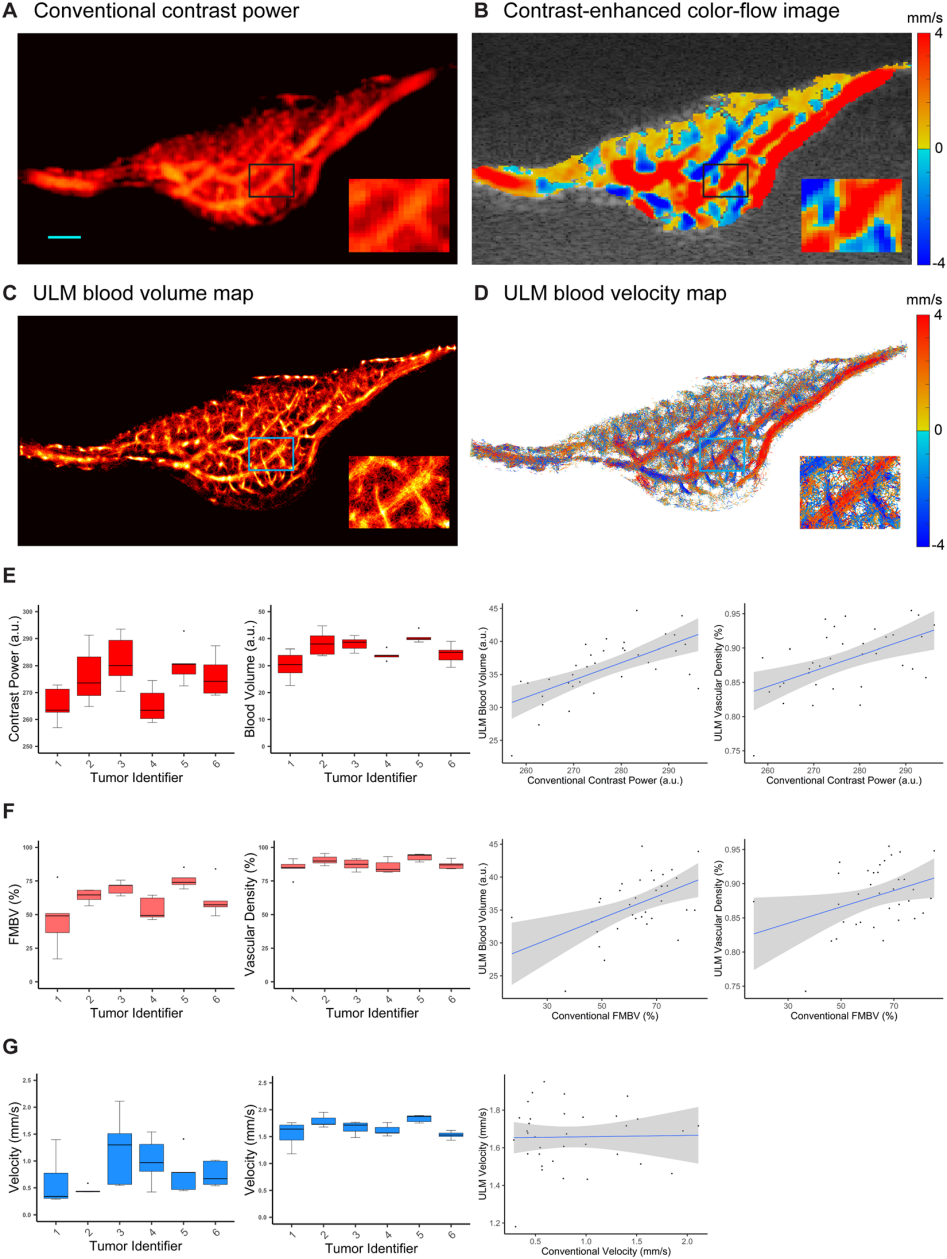
Comparison between diffraction limited CEUS and ULM (**A**) Contrast-enhanced power images demonstrate that CAM-engraft Renca tumors are highly vascularized through-out the entire tumor volume. (**B**) Contrast-enhanced color-flow imaging exhibited a mosaic pattern of alternating blood flow velocity directions, with the majority of faster flow typically present in the tumor periphery. (**C**) Super-resolution microbubble localization maps demonstrated improved resolution of small vessels, while retaining the vascular features of the larger feeding vessels. (**D**) The microvessel blood-flow velocity images disambiguated some of the velocity information present in the conventional color-flow images, particularly for smaller vessels. The interconnectedness of flow direction is more apparent. (**E**) Both conventional power images and super-resolution localization maps demonstrated similar trends in their estimate of blood volume for each tumor, with a high significant correlation (R = 0.83, p = 8.7 × 10^−7^, N = 30, Spearman correlation) (**F**) Likewise, the correlation between the conventional estimate of FMBV and ULM vascular density was significant, but only moderate in magnitude (R = 0.59, p = 0.0006, N = 30, Spearman correlation) (**G**) Estimates of mean flow velocity between the two image processing techniques were not found to be significant (R = 0.036, p = 0.85, N = 30, Spearman correlation).

Conventional contrast power (Fig. 3A) and contrast-enhanced color-flow images (Fig. 3B) were generated from the same imaging dataset for the purpose of a direct side-by-side comparison to super-resolution processing. Conventional contrast power (Fig. 3A) was consistent with super-resolution microvascular imaging (Fig. 3C) in demonstrating that the CAM Renca tumors were highly vascularized. The contrast power estimates of blood volume were significantly correlated to ULM estimates of blood volume (Fig. 3E, R = 0.83, p = 8.7 × 10^−7^, N = 30, Spearman correlation) and vascular density (R = 0.59, p = 0.0006, N = 30, Spearman correlation). The arteriole/venule-scale vessels on conventional imaging could also be identified on ULM images, while the diffusive appearance of the microvascular bed was better resolved as small vessels. Using the contrast power data, we also estimated the fractional moving blood volume (FMBV)^31,36^, which was found to be significantly correlated to ULM estimates of vascular density (Fig. 3F, R = 0.59, p = 0.0006, N = 30, Spearman correlation) and blood volume (R = 0.56, p = 0.0017, N = 30, Spearman correlation). Contrast-enhanced color-flow images (Fig. 3B) demonstrated mosaics of alternating blood flow directions and velocities. Super-resolution velocity maps (Fig. 3D) revealed the same mosaic patterning and disambiguated some of the blood flow velocity for smaller vessels. Velocity estimates between the two image processing techniques were not significantly correlated (Fig. 3G, R = 0.036, p = 0.85, N = 30, Spearman correlation), likely in part due to the known poor performance of color-flow imaging relative to power imaging for small vessels and slow flow^37^. Generally, conventional-resolution contrast-enhanced color-flow imaging suffered from a high degree of quantification variability in comparison to ULM velocity estimates. The mean ± standard deviation of the coefficients of variation (COV) for diffraction-limited CEUS measurements of signal power, FMBV, and velocity were 2.9% ± 0.6%, 18.1% ± 15.8% and 45.1% ± 21.3% respectively. The COV for the corresponding ULM measures of blood volume, vascular density, and velocity were 9.6% ± 4.9%, 4.8% ± 1.7%, and 7.2% ± 4.3%, respectively. However, it should be noted that the imaging acquisitions used in this study were optimized for ULM processing and not contrast-enhanced Doppler imaging. The low transmit voltage and sparsity of blood pool scatterers are plausible explanations for the relatively poor performance of diffraction-limited CEUS measurements in this case.

### ULM datasets are rich in both structural and physiological metrics

**Figure 4:**
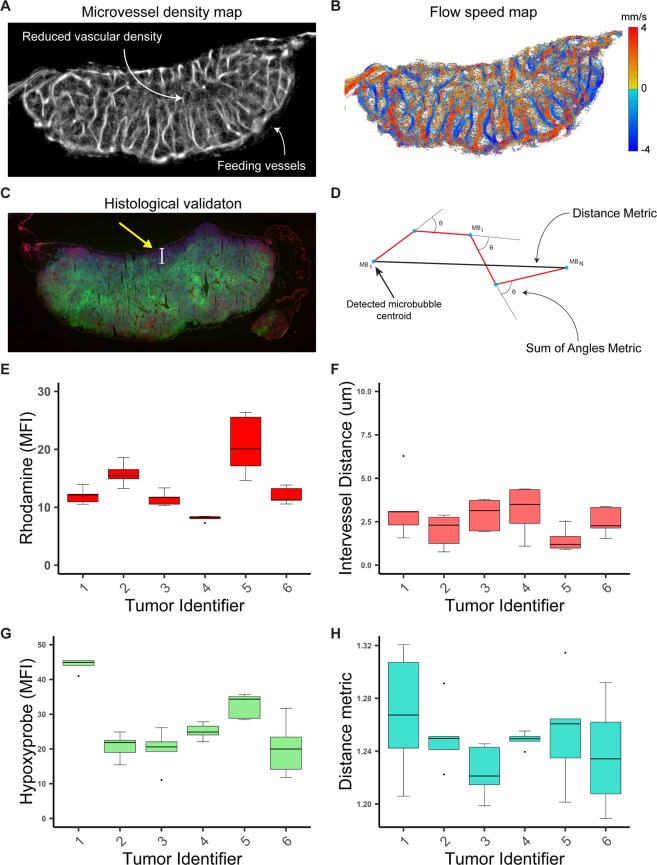
Intratumoral heterogeneity of CAM Renca tumors. (**A**) Tumor microvessel density maps demonstrate a phenotype of lower microvascular density in the center of the tumor, with most of the large feeding vasculature found in the tumor periphery. (**B**) ULM blood flow velocity images corroborate these findings, with slower blood flow velocities found in the center of the tumor and a characteristic mosaic patterning of multidirectional blood flow directions. (**C**) Hypoxyprobe signal demonstrated that the center of this tumor is hypoxic, but showed that the top-most region was well oxygenation (arrow). This may be due to oxygen diffusion from the atmosphere (Supplementary Fig. 1). (**D**) The tortuosity of the tumor microvasculature was calculated using the Distance Metric and Sum of Angle Metric. (**E**) Boxplot of the rhodamine MFI, a histological measure of microvascular density. (**F**) ULM intervessel distance quantifications. (**G**) Boxplot of hypoxyprobe MFI taken as a quantified measure of intratumoral hypoxia. (**H**) The Distance Metric for this tumor cohort.

The accumulated microbubble localization maps offer microvascular structural information for the tumor (Fig. 4A). These can include descriptions of vascular anatomy and morphology, such as metrics for organization, spatial distribution, and tortuosity, along with quantifications of microvascular density and blood volume. Blood volume estimation via microbubble centroid accumulation is the most fundamental of these metrics and is analogous to conventional contrast power. Derived metrics such as intervessel distance and vascular tortuosity can be derived from processed microbubble localization maps. The intervessel distance is informative of the local access of tissue to blood supply, and tortuosity can be indicative of sluggish flow and poorly perfused regions^38^. The calculation of tortuosity metrics such as the distance metric (DM) and sum of angles metric (SOAM) are straightforward given that ULM relies on a strictly intravascular tracer. Likewise, the blood-flow velocity maps (Fig. 4B) provide a rich array of physiological information on the imaged tumor, including perfusion metrics and volume-flow rates. The most fundamental of these is a measurement of the mean blood flow velocity within the tumor. The volume flow rate of the vasculature — that is, the contrast microbubble signal power weighted flow velocity — is a potential measure of the perfusion rate within the tissue.

### CAM Renca tumors exhibit intratumoral vascular heterogeneity

ULM processing revealed intratumoral vascular heterogeneity for much of this tumor cohort. Tumor microvessel density maps demonstrated a phenotype reduced vascular density in the central regions of the tumor mass, with the majority of large feeding vasculature found in the tumor periphery (Fig. 4A). Likewise, blood flow velocity was reduced in the center of the tumor, as evidenced in the ULM flow speed maps (Fig. 4B), which show a characteristic mosaic pattern of flow direction and speed. These features taken in combination may indicate that the center of this tumor would be hypoxic, and prediction that was suggested with histological sections from the same tumor region (Fig. 4C). Generally, the center of each tumor mass was found to be the most hypoxic, which is consistent with the hypothesis of diffusion limited (chronic) hypoxia^39^. One notable disparity, however, between the oxygenation map and the histological comparison, is for the top-most region of the tumor (arrows). One plausible explanation is that this region of the tissue, by being exposed to environmental atmosphere, is passively provided with oxygen. This conclusion is supported by our hypoxia findings for the tumor that died prior to the tumor exaction procedure (Supplementary Fig. 1). We used both DM and SOAM as established surrogate measures of the tortuosity seen in tumor microvasculature, which is characteristically chaotic and topographically disordered. The DM and SOAM were calculated as in^38^, which is depicted diagrammatically in Fig. 4D. Fluorescent histology served as our reference standard for tumor vascularization and hypoxic status. Quantifications of the mean fluorescent intensity (MFI) are reported as box-plots in Fig. 4. The MFI of the rhodamine signal demonstrated a high degree of intratumoral heterogeneity (Fig. 4E), and it was found to be significantly correlated with ULM intervessel distance (Fig. 4F). Likewise, the MFI of hypoxyprobe and corresponding hypoxic percentage of tumor area were also highly variable between individual tumors in the cohort (Fig. 4G). The DM was found to be significantly correlated to tumor hypoxia (Fig. 4H), however no established gold-standard exists to validate this observation.

### Histological assessment correlates with ULM metrics

**Figure 5:**
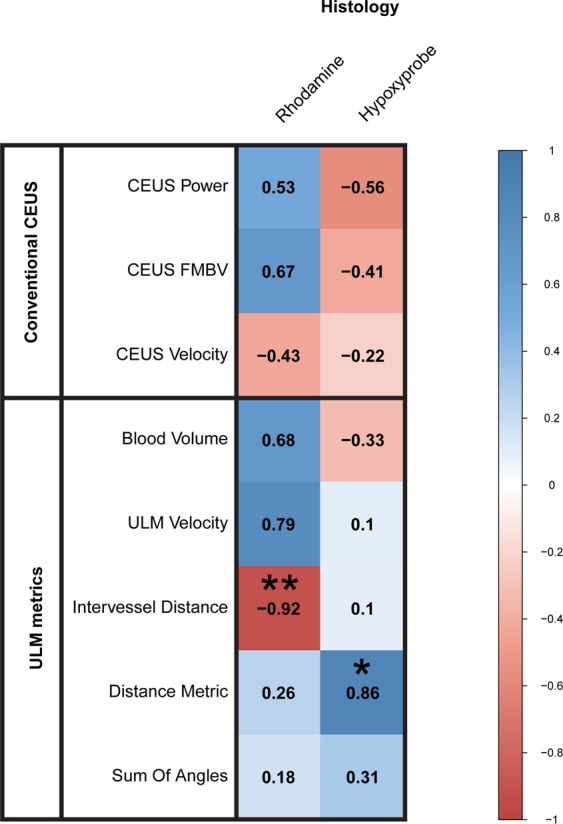
Correlation to fluorescent histology. Correlation matrix of histological measurements in comparison to the vascular metrics derived from ULM imaging (Pearson’s correlation coefficient (R)). Notable findings include a significant negative correlation between rhodamine and ULM intervessel distance (R = −0.92, p = 0.01, N = 6), and a significant positive correlation between hypoxyprobe signal and the Distance Metric (R = 0.86, p = 0.03, N = 6). Significant correlations are denoted with asterisk (*p < 0.05, **p < 0.01).

We constructed a correlation matrix of histological measurements in comparison to the quantified metrics derived from ULM imaging in order to better visualize the correlational relationships between each metric (Fig. 5**)**. This correlation matrix lists the Pearson’s correlation coefficient within each box, with statistical significance denoted with asterisk (*p < 0.05, **p < 0.01). Notable findings include significant correlations between rhodamine MFI and ULM intervessel distance (R = −0.92, p = 0.01, N = 6). In comparison, the correlation between conventional contrast FMBV and rhodamine MFI was moderate (R = 0.67, p = 0.14, N = 6), as was the correlation between conventional contrast power and rhodamine MFI (R = 0.53, p = 0.28, N = 6). A significant positive correlation was found between DM and the hypoxyprobe quantification (R = 0.86, p = 0.03, N = 6), implying that highly chaotic microvasculature results in poor tissue oxygen delivery.

## Discussion

We evaluated the utility of ULM imaging on a chicken embryo chorioallantoic membrane tumor xenograft model of renal cell carcinoma. Consistent with other research groups^26,28,29^, we demonstrated that ULM provides a dataset that is rich in both structural (anatomy, morphology) and physiological (velocity, flow rate) metrics at the scale that is approaching the microvascular level in tumors. In particular, the assessment of vascular tortuosity and corresponding correlation to intratumoral hypoxia are enticing, given that tissue hypoxia has been shown to be predictive of poor outcomes across a broad spectrum of disease states. Within the context of oncology, intratumoral hypoxia has been correlated to increased disease aggressiveness^40^, metastatic potential^14^, and clinical resistance(s) to cytotoxic therapies, including chemotherapy^8^, radiation therapy^9^, and immunotherapy^10^. We found that the ULM metric of intervessel distance was significantly correlated to histological measurements of microvascular density as quantified with rhodamine MFI (Pearson’s correlation coefficient of R = −0.92, p = 0.01, N = 6). Somewhat surprisingly, the ULM blood volume metric was not significantly correlated to rhodamine MFI, likely due to the dependence on microbubble centroid counts. One measure of vascular tortuosity, DM, was significantly correlated with hypoxyprobe quantifications (R = 0.86, p = 0.03, N = 6) indicating that ULM may be sensitive to the vascular heterogeneity typical of tumor-induced angiogenesis^26^. Conventional contrast techniques, such as contrast power, FMBV, and color flow imaging, did not perform as well as ULM imaging on this tumor cohort, potentially due to the low transmit voltage and sparsity of blood pool scatterers.

ULM outperforms conventional contrast imaging in terms microvascular detection and vessel localization sensitivity for several reasons. By providing an imaging resolution that is beyond the diffraction limit of ultrasound, we are not bounded by the inherent trade-off between imaging resolution and imaging depth. The bipartite graph-based microbubble pairing and tracking algorithm^34^ and Kalman filter smoothing^35^ used in this study provides high-confidence microbubble trajectories through strict velocity and acceleration exclusion criteria. It is unclear if ULM outperforms diffraction-limited CEUS for flow parameter estimation given the conflicting requirements for optimal scatterer density and transmit voltages. Although not expressly tested in the current study, it is reasonable to assume that ULM would also outperform non-contrast imaging techniques, such as power Doppler Ultrasound Microvessel Imaging^31^, given that conventional contrast-enhanced ultrasound is more sensitive to blood flow than non-contrast Doppler techniques. However, an important limitation of ULM in comparison to conventional contrast and non-contrast ultrasound imaging is the requirement for a longer image accumulation time. This is particularly problematic for free-breathing animal imaging and clinical translatability, where respiratory and tissue motion artifacts can considerably reduce the fidelity of microvascular reconstruction. ULM is also limited in its temporal resolution due to this accumulation requirement, and may fall short when dynamic blood flow information is needed. This limitation warrants further development of the ULM technique.

We relied on a histological standard to correlate with the ULM metrics developed for ULM imaging. However, a substantial limitation of this approach is that no true gold-standard has been established for the dynamic metrics proposed in this manuscript. This is particularly pertinent given that microbubble motion was detected, and velocities estimated, from two-dimensional images. Thus the velocity of flow measurements acquired in this study may be underestimated due to out-of-plane microbubble motion, and the degree of this underestimation will depend on the flow direction of a complex three-dimensional vascular structure. High volume-rate three-dimensional ultrasound imaging potentially offers a technique to disambiguate out-of-plane microbubble motion, but such high volume-rate solutions are technically challenging to implement and expensive, making 3D ULM a field of ongoing research. ULM uniquely provides a high degree of microvascular fidelity at clinically relevant penetration depths that has never been achieved before non-invasively. Deep tissue microvessel-scale blood velocity and tissue volume flow-rate cannot be directly validated by either histology or competing imaging modalities. Morphometric quantifications, such as vascular tortuosity, could potentially be validated with vascular-casting contrast-enhanced micro-CT on fixed tissues;^41^ however, our preliminary results found that this method is technically challenging to implement on the CAM tumor model (Supplementary Fig. 2). ULM also has the distinct advantage that, except for an intravascular injection, it is a non-invasive imaging modality that is free of ionizing radiation, which warrants frequent clinical follow-up exams. This allows for study designs that track longitudinal vascular changes, in which all ULM metrics can use an individual tumor as its own reference point to serve as an internal control. This, in turn, would permit the exploration of a vascular normalization hypothesis^42^ and the evaluation of combinational therapies. By tracking microvascular phenotypes (for example: vessel tortuosity), an investigator could pin-point the ‘vascular normalization’ time window that is hypothesized to be critical to the synergistic interaction between anti-angiogenic therapies and conventional cytotoxic treatments. Further investigation is required to examine the robustness of vascular tortuosity metrics, such as DM, for large animal and clinical imaging scenarios that exhibit substantial motion artifacts, as respiratory and/or tissue motion could be erroneously construed as chaotic microbubble trajectories.

The reliance on fluorescent histology is not without its caveats, as there are multiple causes for quantification variability. Rhodamine lectin (*lens culinaris* agglutinin) binds to glycoproteins located in the luminal space of actively perfused vessels when injected intravascularly. This allows for the visualization of microvascular morphology and the quantification of relevant capillary network features such as microvascular density. However, this method is susceptible to injection variability, vascular delivery, and photo-bleaching^43^. Furthermore, by binding to the vascular glycocalyx, lectin is only reporting the location of the outer vascular lumen and therefore under-reports the blood volume contribution of larger vessels (see the arrow in Fig. 1E). We co-injected lectin with hypoxyprobe, which readily diffuses into all tissues but only binds to cells with an oxygen tension less than 10 mmHg, to permit the immunochemical detection of hypoxia gradients within the tissue of interest. Given that the fluorescent intensity of hypoxyprobe does not strictly correlate with the degree of hypoxia within a tumor, we applied a threshold to calculate the percentage of tumor area that was positive for hypoxyprobe signal. As seen with one of our tumors (Supplementary Fig. 1), there is also the potential for false positive signals due to the tumor extraction procedure. Furthermore, this technique can be susceptible to acute — or perfusion limited — hypoxia due to transient changes in vascular supply. The relative importance of diffusion limited versus perfusion limited hypoxia on cancer outcomes is currently unknown^39^. Based on the current findings a future study with a larger N number is warranted to confirm that the proposed metrics are robust and reproducible. With all limitations taken into consideration, fluorescent histology can be considered at best to be a semi-quantitative standard, especially when compounded with the difficulty of matching ultrasound imaging planes with histological cross-sections.

We have shown that ULM, by providing non-invasive *in vivo* microvascular structural and physiological information, can potentially be leveraged to indirectly inform tissue oxygenation status along with other critical hemodynamic metrics. Therefore, ULM has the potential to be a crucial clinical imaging modality for the diagnosis and therapy monitoring of solid tumors.

## Methods

### Ethics approval

As avian embryos are not considered to be “live vertebrate animals” according to the NIH PHS policy, no IACUC approval was necessary to conduct the chicken embryo experiments presented in this study.

### Preparation of reagents

Hypoxyprobe powder (pimonidazole hydrochloride, Chemicon) was dissolved in deionized water to produce a 116 mg/mL stock solution. The target dosage of hypoxyprobe in mice is 50 mg/kg, equating to 23.8 µL of the stock hypoxyprobe solution for each chick embryo (approximate weight of an egg is 55 g). Rhodamine lectin [*lens culinaris* agglutinin^44^] was diluted at a 1 to 10 ratio with PBS and combined with hypoxyprobe in preparation for intravascular injection. A stock Hoechst solution was diluted with PBS at a 1 to 1000 ratio in PBS for histological staining.

### Cell culture

The cell culture methods and CAM engraftment procedures were followed as detailed in^31^. Briefly, the Renca cell line (ATCC CRL-2947) was obtained from the American Type Culture Collection Inc. (Bethesda, MD). Renca cells were maintained in RPMI 1640 media (Wisent, QC) supplemented with 10% FBS (Hyclone, UT), sodium pyruvate (1 mM), non-essential amino acids (0.1 mM), and L-glutamine (2 mM). Cells were sub-cultivated when above 80% confluency at a 1 to 5 ratio. All cells were kept in a 37 °C humidified incubator with a 5% CO_2_ atmosphere.

### Cell line engraftment into the CAM

In preparation for the day of engraftment (ninth day of embryonic development, EDD-9) Renca cells were grown to 80% confluency or greater and trypsinized (0.05% Trypsin-EDTA, Wisent) to detach from cell culture flasks. Detached cells were centrifuged for 5 minutes at 300 g. The resulting cell pellet was re-suspended in 10 mL of PBS and a cell count was generated using the Countess cell counter (Life Technologies). Cells were then pelleted again (300 g for 5 minutes). The cell pellet was mixed with Matrigel (BD Bioscience), resulting in a mixture of 4 × 10^5^ cells per 10 µL inoculation dose. The cell-Matrigel mixture was kept on ice until implantation into the CAM.

Fertilized chicken eggs were obtained from Hoover’s Hatchery (Rudd, IA) and transferred into plastic weight boats on EDD-4. On EDD-9, a 5-mm disk of autoclaved Whatman No.1 filter paper was used to scrape the surface of the CAM, and 10 µL of the cell-Matrigel mixture was added into the area. A total of 7 chicken embryos were prepared with Renca cell line tumors for this study.

### Ultrasound imaging protocol

All ultrasound imaging was performed with a Verasonics Vantage 256 ultrasound system (Verasonics Inc., Kirkland, WA) equipped with a high-frequency linear array transducer (L35-16vX, Verasonics Inc.). A center frequency of 25 MHz was used to provide a plane-wave ultrasound transmission. Imaging was performed using a 15-angle plane-wave compounding (−7° to 7°, 1° increment) at a frame rate of 500 Hz (i.e., 500 compounded frames per second), and a transmit voltage of 5 volts. The voltage was empirically determined to provide minimal native blood signal in the CAM model while still maintaining a good signal-to-noise ratio from microbubbles. Commercially available microbubbles (Bracco Lumason) were reconstituted with 1 mL sterile injection saline, yielding a solution with approximately 1.8 × 10^9^ microbubbles/mL. CAMs were injected with a single 70 µL bolus of microbubbles (1.4 × 10^8^ microbubbles total), into a small vein on the CAM surface (Fig. 1A), with no saline flushing. Injections were performed with a glass capillary needle (B120-69-10, Sutter Intruments, Novato, CA, USA), that was pulled using a PC-100 glass puller (Narishige, Setagaya City, Japan), fitted onto Tygon R-3603 laboratory tubing and an 18Gx1.5” BD PrecisionGlide needle with 1 mL syringe. Imaging acquisitions were acquired at the microbubble concentration plateau after the bolus peak. There were a total of 5 imaging planes sampled throughout each tumor mass. Each of the five tumor planes was imaged using 5 separate acquisitions of 720 frames each, for a total acquisition length of 3600 frames (7.2 seconds) per plane. Acquisitions were stored as in-phase/quadrature (IQ) demodulated data sets for post-processing in MATLAB.

### Ultrasound signal processing

A spatiotemporal SVD-based clutter filter was applied to the IQ data to suppress tissue signals as in^31,33,34^, which is depicted in Fig. 2B. Specifically, each 720 frame IQ dataset was reshaped into a 2D Casorati matrix and a SVD decomposition was performed to separate singular values. The low-order singular values represent predominantly tissue signal, thus a threshold cutoff value was applied to zero out these signal components. This threshold was determined by the decay rate of the singular value curve^31,45^, and was set to filter out the first 8 singular values. As noted in^31^, noise suppression via a high order threshold on the SVD curve was unnecessary for CAM imaging, likely due to the shallow imaging depth. An inverse SVD was then performed on the filtered dataset and the new Casorati matrix was reshaped into a 720 frame cineloop. The resulting clutter-filtered imaging dataset was used to generate contrast-enhanced power and color-flow images (Fig. 3A,B) as well as super-resolution ULM microbubble localization maps and velocity maps (methods detailed in the next section). For contrast-enhanced power images, we cumulated the power of the microbubble signals along the temporal dimension. Contrast-enhanced color-flow images were calculated by estimating the flow velocity using a well-established 2D autocorrelation technique introduced by Loupas *et al*.^46^. Two quantitative indices we calculated from the tumor cross-sections of power images: the mean power, and the fractional moving blood volume (FMBV) as described by Rubin *et al*.^36^. The average tumor flow velocity was taken as mean of the absolute value of the estimated flow speeds. Tumor regions of interest (ROIs) of the entire tumor slice were generated by manually delimiting Bezier control points surrounding the cross-section of interest and calculating an interpolating spline using Hobby’s algorithm^47^.

### Super-resolution microbubble localization and tracking

The main processing steps used to produce super-resolution ULM images are detailed in Fig. 2, following the procedure outlined in^34^. A noise-equalization profile^48^ was applied to the SVD-filtered IQ imaging volume to improve image quality and equalize microbubble intensity throughout the field-of-view. The data was then spatially interpolated to an isotropic in-plane axial-lateral resolution of 5 µm using 2-D spline interpolation. The point-spread function (PSF) typical for our imaging system was generated using a multivariate Gaussian function, with the full-width at half-maximum for the axial and lateral dimensions of this PSF empirically determined from isolated microbubbles in the imaging plane (Fig. 2D). Although the PSF function for ultrasound imaging is spatially variant, we assumed that there was an invariant microbubble PSF for CAM tumor processing given that the tissue was shallow and had limited attenuation. A 2-D normalized cross-correlation between every frame of the interpolated microbubble signal and the microbubble PSF was performed across the entire imaging field of view to identify potential microbubble locations. An empirically determined threshold was then applied to reject pixels with a cross-correlation coefficient less than 0.6, yielding isolated microbubble pixel regions^34^. Cross-correlation peaks were then localized using a regional maximum search with the “imregionalmax.m” function in MATLAB, resulting in microbubble centroid maps (Fig. 2E). Microbubbles were then paired frame-by-frame using a bipartite graph-based pairing and tracking algorithm^34^, with a minimum persistence of 10 frames, a maximum velocity of 20 mm/s. This velocity threshold was selected based on the expected flow velocity of chorioallantoic membrane vasculature that is below 200 µm in diameter^49^. The result of one such frame-to-frame pairing is demonstrated in Fig. 2F. Microbubble tracks and flow velocities were then reconstructed using a Kalman-filter based algorithm, with a constraint of a frame-to-frame microbubble trajectory turn angle of less than 90°, to reject unreliable microbubble tracks^34,35^. An example of the final resulting microbubble localization map, which identified vasculature down to capillary scale, is shown in Fig. 2G. The corresponding flow-velocity map is shown in Fig. 2H.

### ULM image analysis

Cross-sectional ROIs of the entire tumor slice were outlined using the Hobby spline method detailed above. ULM blood volume was calculated by accumulating microbubble centroids for the total acquisition length, resulting in intensity weighted microbubble location maps. Vascular density was then estimated from these maps by assigning a binary threshold to these images, where the vascular density percentage was the total number of ‘vascular’ pixels divided by the total ROI area. Inter-vessel distance was calculated by searching within a neighborhood surrounding all the ‘non-vessel’ pixels until the closest ‘vascular’ pixel was identified. The pixel value of the candidate location was then assigned to the Euclidean distance between these two points, where all ‘vascular’ pixels were assigned to a value of zero. Vascular tortuosity was estimated via two established tortuosity metrics, the Distance Metric (DM) and Sum of Angles Metric (SOAM) as detailed in^38^, and demonstrated in Fig. 4D. Briefly, for every detected microbubble track of at least 10 persistent frames the total track length (i.e. the cumulative sum of each microbubble’s frame-to-frame movement) and beginning-to-end distance of each track were used to calculate the DM. The angle between each microbubble centroid location on these tracks was used to calculate the SOAM. The mean blood speed of the flow velocity images was taken as the average of the absolute value of the flow velocity.

### Histological microvascular and hypoxia quantification

We adapted the histological procedures used in^31^ to account for the addition of hypoxyprobe to this study. After ultrasound imaging, tumor bearing CAMs were injected with 100 µL of the hypoxyprobe and rhodamine lectin mixture that was detailed above. Embryos were then returned to the incubator to allow the lectin-hypoxyprobe mixture to circulate for 30–60 minutes. After circulation, tumors were excised with dissection scissors, washed with PBS, embedded in optimal cutting temperature compound (O.C.T. Sakura), and then flash frozen using an isopentane filled beaker that was stored in crushed dry ice. Tumor blocks were serially sectioned using a Leica CM1860 cryostat at a thickness of 7 µm.

### Immunohistochemistry

Histological sections were blocked in 2% bovine serum albumin (BSA) and 10% fetal bovine serum (FBS) for one hour at room temperature, followed by incubation with the primary antibody (0.6 µg/mL anti-pimonidazole mouse IgG1 monoclonal antibody 4.3.11.3) for one hour at room temperature. Sections were then rinsed three times with PBS and covered with a Hoechst solution for 10 minutes at room temperature. Sections were then rinsed again with PBS, air dried, mounted with VECTASHEILD antifade mounting medium (VectorLabs), and cover slipped.

Similar to^31^, histological images were acquired and digitized using a Zeiss Axio Scan.Z1 with a CY3 channel exposure of 400 ms, EGFP channel exposure of 75 ms, and a DAPI channel exposure of 3 ms. CZI files were exported as tiff images and analyzed using MATLAB. ROI segmentations of the tumor cross-sections were performed using manually placed Bezier control points and interpolated using Hobby’s algorithm^47^, as detailed above. Tumor ROIs of the entire tumor cross-section were used to quantify the mean fluorescent intensity (MFI) of the rhodamine lectin stain, as a metric of intratumoral vasculature, and the hypoxyprobe stain, as a measure of tissue oxygenation. The percent tumor area positive for hypoxia was calculated by applying a threshold to exclude hypoxyprobe background fluorescence.

### Statistical analyses

All statistical analyses were conducted in the R programming language^50^. Boxplots and scatter plots were produced using the package ggplot2^51^ and correlation matrices were produced using the package corrplot package^52^. Boxplot whiskers extend to 1.5 times the interquartile range of the data, with data points outside this range considered to be outliers. Unless otherwise noted, correlations were calculated using a linear least squares regression and reported as a Pearson’s correlation coefficient. Correlations between ultrasound and histological were performed on the average of repeated measures to better conform to the Independent and Identically Distributed (IID) assumption. Confidence intervals for Pearson’s correlation coefficients were calculated using a Fisher’s z’ transformation. Statistical significance was tested using a two-way t-test, where a p < 0.05 was considered to be statistically significant.

## Supplementary information

Supplementary information.

Supplementary Figure 1.

Supplementary Figure 2.

## Data Availability

The data that support the findings of this study are available from the corresponding authors on request.
