# Supplementary material for: Ultrasound localization microscopy of renal tumor xenografts in chicken embryo is correlated to hypoxia
Source: Sci Rep. 2020 Feb 12;10:2478. doi: 10.1038/s41598-020-59338-z (PMC7015937; doi:10.1038/s41598-020-59338-z)
Supplement: Supplementary file 1 — Supplementary information. [file 41598_2020_59338_MOESM1_ESM.docx]

# Ultrasound localization microscopy of renal tumor xenografts in chicken embryo is correlated to hypoxia

Matthew R. Lowerison^1,2,3,§^, Chengwu Huang^3,§^, Fabrice Lucien^4^, Shigao Chen^3,*^, Pengfei Song^1,2,3,*^

^1^ Beckman Institute for Advanced Science and Technology, University of Illinois at Urbana-Champaign, Urbana, IL

^2^ Department of Electrical and Computer Engineering, University of Illinois at Urbana-Champaign, Urbana, IL

^3^ Department of Radiology, Mayo Clinic College of Medicine and Science, Mayo Clinic, Rochester, MN

^4^ Department of Urology, Mayo Clinic College of Medicine and Science, Mayo Clinic, Rochester, MN

^§^ These authors contributed equally to this work.

*Corresponding Authors:

Dr. Pengfei Song

Department of Electrical and Computer Engineering

Beckman Institute for Advanced Science and Technology

University of Illinois at Urbana-Champaign

405 N. Mathews Ave.

Urbana, IL 61801

Email: [songp@illinois.edu](mailto:songp@illinois.edu)

Dr. Shigao Chen

Department of Radiology

Mayo Clinic

200 First Street SW

Rochester, MN 55905

Email: Chen.Shigao@mayo.edu

# Supplementary Figure Legends

## Figure S1 | Passive oxygen diffusion in dead tumor

Hypoxyprobe staining taken from the chicken embryo that died during the experiment revealed a distinct boundary for de-oxygenated tissue (white line). This implies that oxygen passively diffuses into the top of the CAM tumor xenograft model, which represents a potential source of bias.

## Figure S2 | Microfil injections of CAM vasculature

(**A**) We attempted Microfil injections into the CAM vasculature to permit the use of contrast-enhanced micro-CT as a gold-standard for the ULM metrics explored in this manuscript. However, these injections proved to be technically challenging, with incomplete vascular perfusion, and therefore was not useful as a standard in this study. (**B**) A magnified view of a region of interest (outlined in red) demonstrates that several CAM surface vessels were not perfused with Microfil.
