# Supplementary figures and images for: Ultrasound localization microscopy of renal tumor xenografts in chicken embryo is correlated to hypoxia
Source: Sci Rep. 2020 Feb 12;10:2478. doi: 10.1038/s41598-020-59338-z (PMC7015937; doi:10.1038/s41598-020-59338-z)

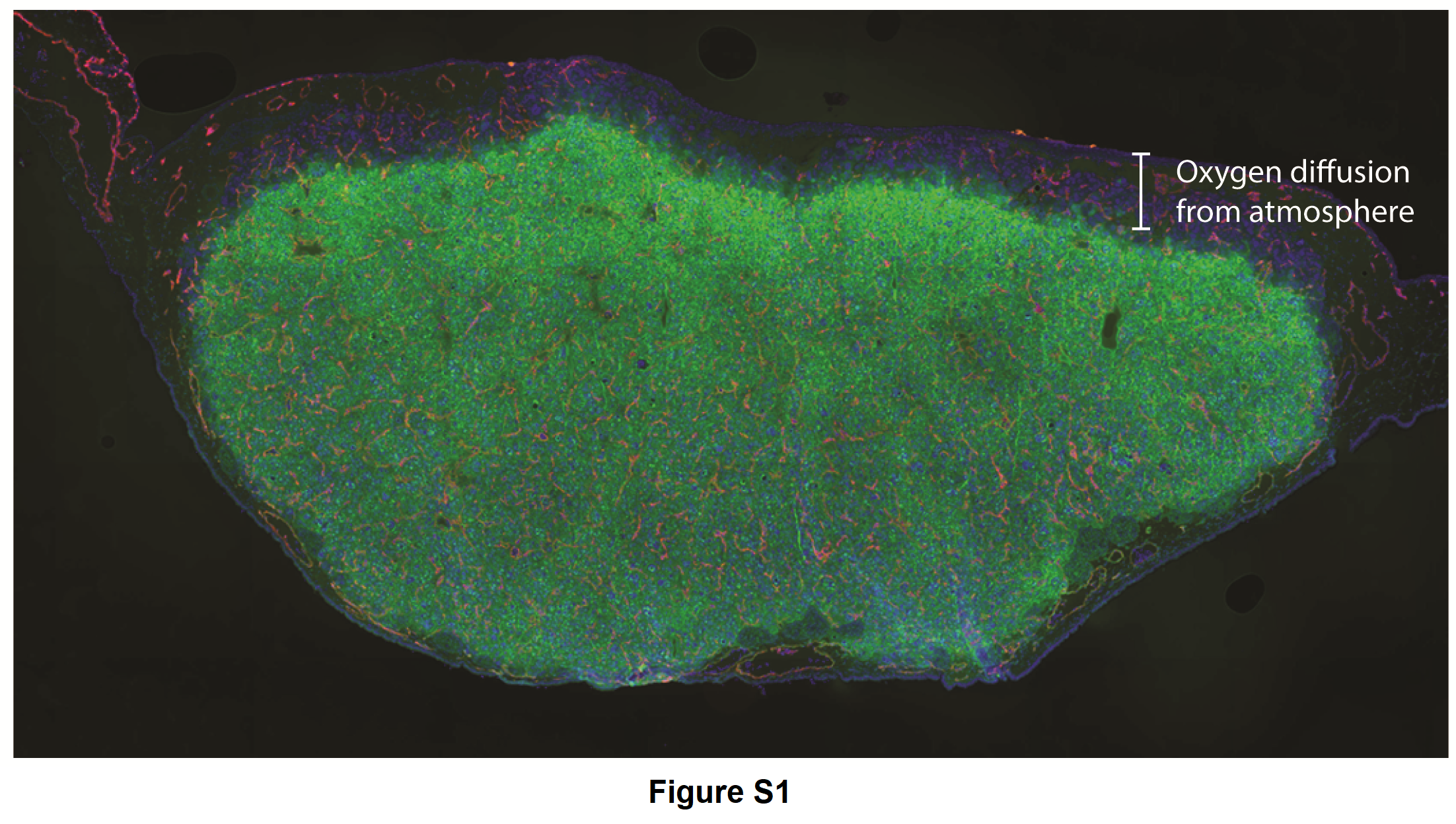

Supplement: Supplementary file 2 — Supplementary Figure 1. [file 41598_2020_59338_MOESM2_ESM.tif]

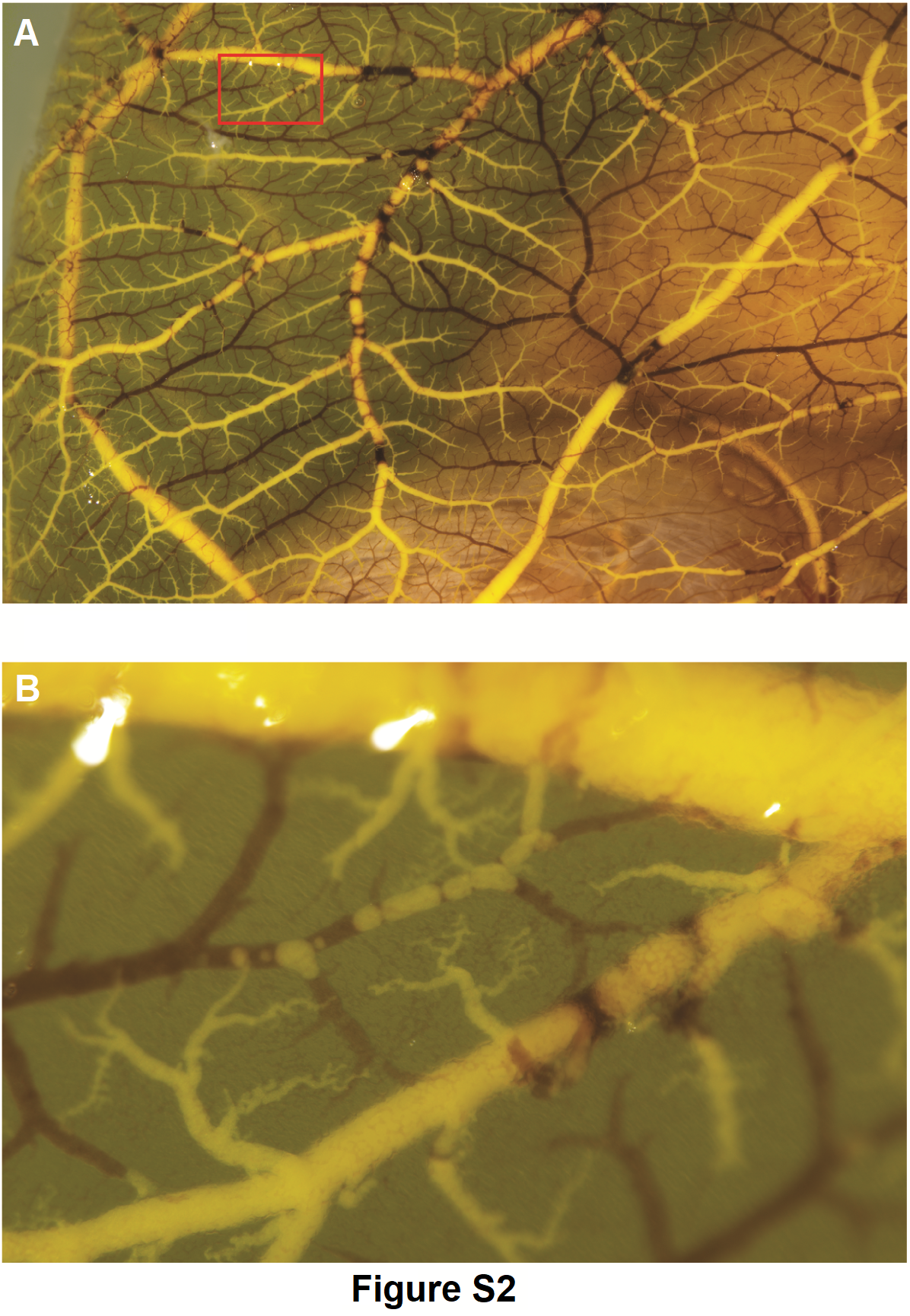

Supplement: Supplementary file 3 — Supplementary Figure 2. [file 41598_2020_59338_MOESM3_ESM.tif]
